# Ketamine induces a robust whole-brain connectivity pattern that can be differentially modulated by drugs of different mechanism and clinical profile

**DOI:** 10.1007/s00213-015-3951-9

**Published:** 2015-05-19

**Authors:** R. Joules, O. M. Doyle, A. J. Schwarz, O. G. O’Daly, M. Brammer, S. C. Williams, M. A. Mehta

**Affiliations:** Department of Neuroimaging, Institute of Psychiatry, Psychology & Neuroscience, Kings College London, De Crespigny Park, London, SE5 8AF UK; Eli Lilly and Company, Lilly Corporate Center, Indianapolis, IN 46285 USA; Department of Psychological and Brain Sciences, Indiana University, Bloomington, IN USA

**Keywords:** Glutamate, NMDA receptor, fMRI, Drug discrimination, Imaging, Schizophrenia, Thalamus, Connection

## Abstract

Ketamine, an N-methyl-D-aspartate receptor (NMDAR) antagonist, has been studied in relation to the glutamate hypothesis of schizophrenia and increases dissociation, positive and negative symptom ratings. Ketamine effects brain function through changes in brain activity; these activity patterns can be modulated by pre-treatment of compounds known to attenuate the effects of ketamine on glutamate release. Ketamine also has marked effects on brain connectivity; we predicted that these changes would also be modulated by compounds known to attenuate glutamate release. Here, we perform task-free pharmacological magnetic resonance imaging (phMRI) to investigate the functional connectivity effects of ketamine in the brain and the potential modulation of these effects by pre-treatment of the compounds lamotrigine and risperidone, compounds hypothesised to differentially modulate glutamate release. Connectivity patterns were assessed by combining windowing, graph theory and multivariate Gaussian process classification. We demonstrate that ketamine has a robust effect on the functional connectivity of the human brain compared to saline (87.5 % accuracy). Ketamine produced a shift from a cortically centred, to a subcortically centred pattern of connections. This effect is strongly modulated by pre-treatment with risperidone (81.25 %) but not lamotrigine (43.75 %). Based on the differential effect of these compounds on ketamine response, we suggest the observed connectivity effects are primarily due to NMDAR blockade rather than downstream glutamatergic effects. The connectivity changes contrast with amplitude of response for which no differential effect between pre-treatments was detected, highlighting the necessity of these techniques in forming an informed view of the mechanistic effects of pharmacological compounds in the human brain.

## Introduction

The N-methyl-D-aspartate receptor (NMDAR) antagonist ketamine induces glutamatergic dysfunction in healthy humans which can lead to the acute onset of psychomimetic symptoms resembling schizophrenia (Krystal et al. 1994; Goff and Coyle 2001; Corlett et al. 2011). Whilst not a phenocopy of the disorder, ketamine causes an increase in both positive and negative symptom scores as well as impairing cognition (Dsouza et al. 1994; Honey et al. 2008). Hence, it has been widely used to study the role of glutamatergic dysfunction in relation to schizophrenia (Lahti et al. 1995).

Ketamine has been shown to elicit robust and reliable effects on the blood oxygen level-dependent (BOLD) signal in healthy volunteers (Deakin et al. 2008; De Simoni et al. 2013; Driesen et al. 2013). To date, these BOLD effects have been primarily studied in terms of their amplitude (Deakin et al. 2008; De Simoni et al. 2013; Doyle et al. 2013a). Moreover, pre-treatment with compounds expected to reduce glutamate release has been demonstrated to attenuate the ketamine-induced BOLD response (Deakin et al. 2008; Doyle et al. 2013a).

Previously, we demonstrated that acute pre-treatment with clinically effective doses of either lamotrigine or risperidone attenuated the BOLD response to ketamine infusion in healthy volunteers via different mechanisms of action (Doyle et al. 2013a). Lamotrigine, an anticonvulsant, acts pre-synaptically through sodium ion channel modulation, attenuating glutamate release directly (Large et al. 2005). In contrast, risperidone, an atypical antipsychotic, is thought to alter cortical glutamate levels pre-synaptically through 5-HT_2A_ receptor antagonism, indirectly reducing glutamate release (Meltzer et al. 2011). Moreover, risperidone has been shown to potentiate NMDARs directly (Konradsson et al. 2006) providing an additional potential mechanism of action to attenuate the ketamine-evoked effect.

Acute NMDAR blockade has been shown to alter the functional connectivity of several neural systems in both rodents (Gass et al. 2013) and humans (Niesters et al. 2012; Driesen et al. 2013). The mechanism by which this is achieved is not understood. It is presumed that the downstream effects on the glutamate system drive connectivity changes, but evidence for this is lacking.

Here, we address the question of how an acute ketamine infusion affects the pattern of whole-brain connectivity and how this is altered through different modulatory mechanisms using risperidone and lamotrigine as pharmacological probes. If purely a result of direct NMDA blockade, we hypothesise that any changes in connectivity will be insensitive to the effects of lamotrigine whilst attenuated by risperidone. We would interpret shared effects by both lamotrigine and risperidone as resulting from the presumed attenuation of glutamate release.

Pattern recognition (PR) techniques have been used to perform single subject inference using whole brain functional connectivity data to model disease states (Craddock et al. 2009; Zhang et al. 2011; Richiardi et al. 2012), cognitive states (Richiardi et al. 2011; Shirer et al. 2012) and age groups (Dosenbach et al. 2010). In the current study, we use whole-brain measures of functional connectivity with PR techniques to identify spatial patterns of whole-brain connectivity underlying the effects of acute ketamine and to investigate the extent of the effect of pre-treatment on this induced connectivity pattern. In accord with our BOLD signal amplitude response findings (Doyle et al. 2013a), we expect both compounds to exhibit modulation of the ketamine-induced change. We predict that the extent and pattern of each compound’s modulatory effect will provide insight into the neuronal systems underlying the ketamine effect in the human brain.

## Materials and methods

### Participants

Twenty right handed male volunteers were recruited for this double blind, placebo controlled, cross-over design study, approved by the Wandsworth Research Ethics Committee (09/H0803/48). Participants provided written informed consent and were screened for any history of physical, neurological and psychiatric illness. Further exclusion criteria included MRI-incompatibilities, illicit drug abuse and excessive alcohol, cigarette or caffeine consumption, in addition to normal ranges on standard haematology and biochemistry assessments. Further details are available in Doyle et al. (2013a). Twenty participants passed screening and were entered into the study, of which four withdrew, one due to fainting upon cannulation in session 1, one experiencing nausea at session 3 and two participants were withdrawn at session 2 and session 3 due to violating the lifestyle guidelines. Thus, 16 participants (range = 20–37; mean = 25.8; SD = 5.7) completed all sessions in this study.

### Experimental design

**Fig. 1:**
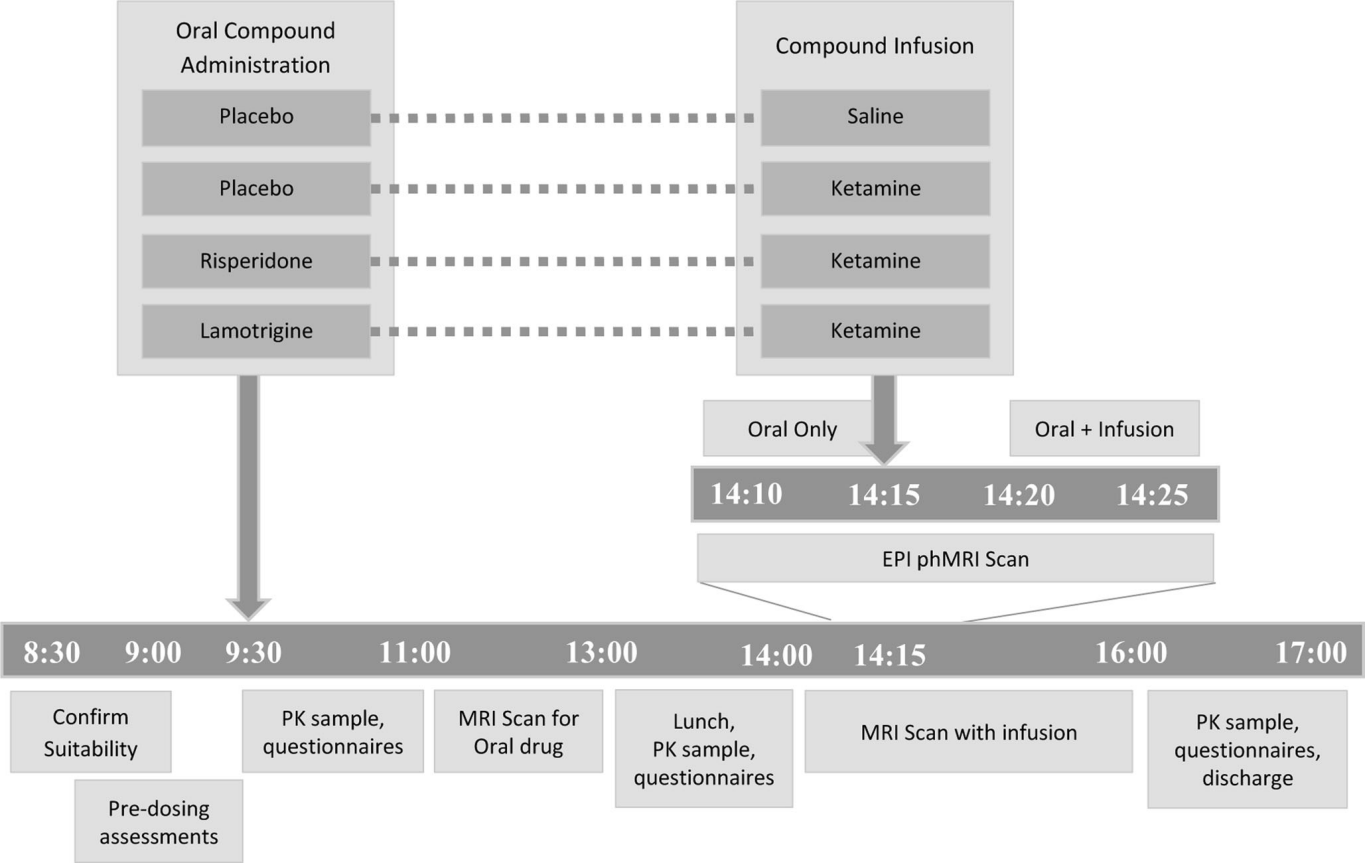
Timeline for each experimental sessions by time of day. Four compound combinations were administered in four separate sessions separated by a period of at least 10 days. Participants received an oral pre-treatment before receiving an intravenous infusion of ketamine or saline with the administered compound combinations of placebo-saline, placebo ketamine, risperidone-ketamine and lamotrigine-ketamine. 156 × 99mm (300 × 300 DPI)

Data were collected over four sessions, each separated by a period of at least 10 days. During each session, participants received a single oral dose of either lamotrigine (300 mg), risperidone (2 mg) or placebo (ascorbic acid; two sessions). This was followed by an intravenous infusion of either saline or ketamine, to a target plasma level of 75 ngml^−1^, timed to coincide with the approximate expected time of maximum plasma concentration of both compounds. The administered pre-treatment and infusion combinations were placebo and saline, placebo and ketamine, risperidone and ketamine and lamotrigine and ketamine. The compound combinations and session timelines are illustrated in Fig. 1. Imaging procedures for risperidone and lamotrigine sessions were undertaken during the broad maximum plasma exposure between 1 h 30 m post-dose (oral drug alone) and 4 h 15 m post-dose during ketamine infusion. At 30 min, 1, 1.5, 4 and 8 h following the administration of the oral compounds, blood samples were taken to measure risperidone and lamotrigine concentrations in plasma.

### Compound infusion

A sub-anaesthetic dose of racemic ketamine was administered intravenously to achieve a target plasma level of 75 ngml^−1^, adjusted for the participant’s weight and height measured at each session. Intravenous administration was achieved using a Graseby 3400 pump based on the Clements 250 model and was controlled using Stanpump (http://anesthesia.standford.edu/pkpd). A dose of 0.12 (±0.003 SD) mgkg^−1^ was administered in the first minute followed by a pseudo-continuous infusion of approximately 0.31 mgkg^−1^ h^−1^. Blood samples were taken at 15 and 75 min after the start of the infusion to measure ketamine concentration in the plasma.

### MRI acquisition

MR images were acquired using a 3T GE HDx scanner. Gradient-echo echo-planar imaging (EPI) was used to acquire 450 task-free volumes of 38 near-axial slices over a period of 15 min for each session (3 mm thickness, 0.3 mm inter-slice gap, TE = 30 ms, TR = 2000 ms, FA = 75°, in-plane resolution = 3.3 mm, matrix size = 64 × 64, field of view = 21.1 × 21.1 cm). The infusion of ketamine or saline was administered 5 min into the 15 min scan. Additionally, a high-resolution gradient-echo scan was performed resulting in 43 near-axial slices (3 mm thickness, 0.3 mm inter-slice gap, TE = 30 ms, TR = 2000 ms, FA = 90°, in-plane resolution = 3.3 mm, matrix size = 128 × 128, field of view = 24 × 24 cm).

### Experimental conditions

The 450 volumes of the EPI time series acquired at each session were divided into pre- and post-infusion conditions, defined as the first and last 150 volumes (5 min) of data, respectively. The pre-infusion condition corresponds to the period during which participants had only the oral compound present and the post-infusion condition corresponds to the period 5–10 min after the administration of bolus. We refer to pre- and post-infusion conditions in each compound combination session using the naming scheme described in Table 1.

**Table 1:** Naming convention for each individual drug state in each of the four compound combinations

Name	Description
Placebo (pre-Sal)	Placebo, prior to saline infusion
Placebo (pre-Ket)	Placebo, prior to ketamine infusion
Risperidone	Risperidone pre-treatment prior to ketamine infusion
Lamotrigine	Lamotrigine pre-treatment prior to ketamine infusion
Saline	Saline infusion given after placebo (pre-Sal)
Ketamine	Ketamine infusion given after placebo (pre-Ket)
Lam + ketamine	Ketamine infused given after lamotrigine
Ris + ketamine	Ketamine infused given after risperidone

*Ket* ketamine, *Lam* lamotrigine, *Ris* risperidone, *Sal* saline

### Pre-processing

Imaging data were pre-processed using SPM5 (www.fil.ion.ucl.ac.uk/spm). The BOLD time series were slice-time corrected, realigned to the first volume then to the mean volume, co-registered to the high-resolution structural scan, spatially normalised to the SPM EPI template and smoothed using a Gaussian kernel of 8 mm at full-width half-maximum. The structural images were segmented into grey matter, white matter and cerebrospinal fluid (CSF) tissue types.

Linear regression was used to regress out nuisance signal parameters from the imaging time-series data, specifically the mean CSF signal, mean WM signal and the six motion parameters. The mean and maximum head motion parameters were compared between compound infusion conditions within each session. No differences in motion were observed at *p* < 0.05 (uncorrected). A band pass filter of 0.01–0.1 Hz was subsequently applied to reduce the effect of high-frequency (>0.1 Hz) physiological signals and low-frequency (<0.01 Hz) noise such as scanner drift.

### Connectivity graph generation

#### Parcellation

The pre-processed data were parcellated into regions to facilitate network analysis where each parcellated region represents a node in a graph. The automated anatomical labelling (AAL) atlas (Tzourio-Mazoyer et al. 2002) was selected to parcellate the brain into 116 anatomical nodes due to its prevalence in neuroimaging studies (Zalesky et al. 2010; Braun et al. 2012). A finer scale parcellation would provide increased regional homogeneity but complicates the interpretation of the model (Faria et al. 2012). Node time series were calculated as the mean of voxel time courses in each atlas region. In this manner, the entire brain can be represented as a graph, *G* = (*V*, *E*), where *V* describes the collection of 116 nodes (defined in the AAL Atlas) and *E* the edges between each node pair.

#### Task-free spatiotemporal dynamics

Task-free or “resting state” networks (RSNs) display complex spatiotemporal dynamics. Networks identified from resting state data are highly dependent on the temporal scale used (Hutchison et al. 2012). In order to account for these dynamics, temporal windowing may be applied (Allen et al. 2012). RSNs derived from long windows (>~120 s) allow investigations of the core network and minimise the effects of spurious correlations which occur with brief acquisitions. Furthermore, it has been demonstrated that RSNs can be considered stable when windowing at >~240 s (Van Dijk et al. 2010; Hutchison et al. 2012). To this end, a moving window of length *T* = 120 volumes (240 s) window was applied over the parcellated time series data for each condition in order to compute temporal connectivity.

A tapered window was preferred to a box car in order to minimise overlap between windows and reduce the contribution of any outliers not central to the window. We define the window using the Gaussian window function:

$$ W(t)= \exp \left(-\frac{t^2}{2{s}^2}\right) $$

where *t* takes values {−T/2 .. T/2} and *s* is defined as T/6. This window was applied in increments of 2*s*, the effective length of the window, resulting in eight windowed time-series for each condition.

In the present study, although no specific task was applied during the functional scan, the paradigm likely induces some departure from the “resting state” due to the expectation of drug delivery pre-infusion and putative subjective effects of ketamine following its infusion.

#### Network formulation

**Fig. 2:**
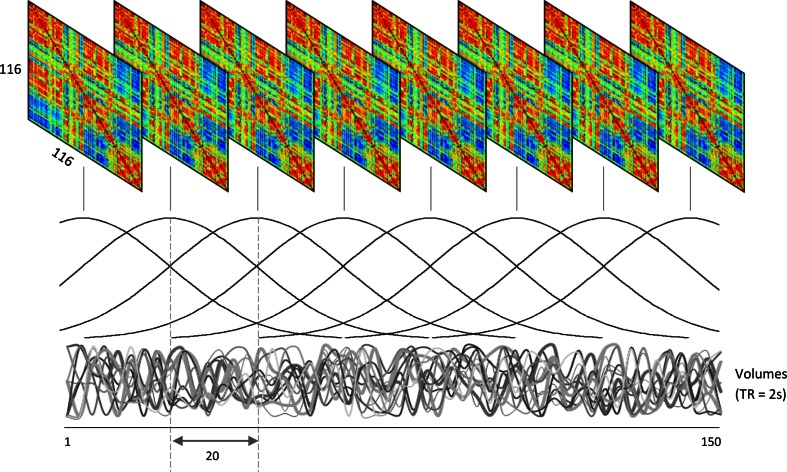
Series of network adjacency matrices obtained using correlations between all node pairs within each window position. Windowing was performed using a moving tapered window of 120 volumes (4 min) applied to each 150 volume (5 min) condition to examine dynamics in the task-free data. 171 × 103mm (300 × 300 DPI)

The edges, *E*, were calculated between all pairs of nodes in V using linear correlation resulting in a series of adjacency matrices for each window position, as illustrated in Fig. 2. In this manner, we express the functional connections between regions across the whole brain whilst accounting for low-frequency network dynamics thought to represent “core” connections (Hutchison et al. 2012).

#### Centrality measure

Using graph theory centrality measures, it is possible to quantify the importance or “connectedness” of node within a graph (Rubinov and Sporns 2010). The most intuitive of these measures is weighted degree centrality which is simply the sum of the weights of all edges impingent on a given node and has been previously demonstrated to be well-suited to elucidate node properties within brain networks (Buckner et al. 2009; Lord et al. 2012). As a local measure, it provides an indication of the connectedness of a brain region with the rest of the brain. Degree centrality was selected for this investigation as it provides an intuitive measure of the overall connectedness of a region. Given the wide spread effects of glutamatergic dysfunction, providing a measure of how the mean connectivity of a region with the rest of the brain is affected. Here, the degree centrality (DC) was calculated for each node, *i*, in each adjacency matrix using:

$$ \mathrm{D}\mathrm{C}(i)=\sum_{j=1}^N{a}_{ij} $$

where *N* is the number of nodes (*N* = 116) and *a*_*ij*_ is the edge weighting between nodes *i* and *j* in the adjacency matrix *A*. Measures at each network slice were normalised by network density to account for inter-subject differences in network densities.

#### Pattern recognition

To perform comparisons between session conditions, Gaussian process classification (GPC) was employed. This binary classification algorithm provides probabilistic predictions of class membership for unseen test data based on a model defined from a set of training data and their respective labels (either +1 or −1). It has been demonstrated to provide robust performance in several neuroimaging studies (Marquand et al. 2010; 2011; Doyle et al. 2013a; Young et al. 2013). For a more detailed account of this technique, please refer to (Rasmussen and Christopher 2006). The subject-wise mean centred DC measures from all window positions in each session condition were used as a feature set for classification, allowing for the comparison of regional network connectedness between conditions.

In order to assess the performance of the classifier, leave-one-out cross validation (LOOCV) was used. This is an iterative process whereby data from all but one participant is used for training; data from the withheld participant is then used for testing. The reported classification accuracy is the mean accuracy value obtained across all LOOCV folds. This accuracy serves to quantify the degree to which the two groups can be separated in terms of their functional connectivity as measured by DC.

The statistical significance of the classification accuracy was assessed by permutation. A distribution of permuted accuracies was generated by re-training the classifier *N*_perm_ times using randomly permuted class labels. The number of times the permuted accuracy was found to be greater than the true accuracy was counted and divided by *N*_perm_ to provide an estimate of the *p* value (here, *N*_perm_ = 1000). As several contrasts were investigated, Bonferroni correction was applied to correct for the effect of multiple comparisons.

Classification was performed using the following two configurations: (1) between PLA + SAL and PLA + KET sessions in order to investigate the effect of ketamine on whole-brain connectivity and (2) using lamotrigine and risperidone pre-treated sessions (LAM + KET and RIS + KET) in contrast to the PLA + KET and PLA + SAL sessions to investigate their modulation of ketamine-induced connectivity.

Ordinal regression using Gaussian processes (ORGP) (Doyle et al. 2013b) was applied to investigate the relationship between experimental conditions, identifying an ordinal trend across classes under the proviso that classes are rank ordered. Classes may be accurately distinguished from one another only if an ordinal progression exists between classes, else poor discrimination may be observed. We refer the reader to Chu and Ghahramani (2005) for further details on this multi-class classification algorithm.

#### Classification maps

Multivariate classification maps (g-maps) were generated for models with significant classification accuracy (identified by permutation tests), allowing for the visualisation of the discriminating pattern of spatial covariance driving the classification (Marquand et al. 2010).

Samples were weighted in relation to their class typicality, resulting in a visualisation of the distribution of the classes relative to one another. For each map, node weights were depicted as spheres positioned in MNI space at the centre of mass for the corresponding AAL regions. The size of these spheres represents the contribution of the node in driving the discriminatory performance. The colour, blue or red, represents the sign of the weights, recalling that the class labels are encoded as either +1 or −1 (e.g. +1 = PLA + KET and −1 = PLA + SAL). A high positive weight implies that the weighted average DC for that node was higher for the class with the positive label +1 (e.g. the PLA + KET class). We note this weighted average is influenced by a combination of the DC value, its variance and the classification parameterisation. The direction of change in node strength between classification groups was confirmed using paired *t* tests. One sample *t* tests were performed on the node deltas between classes for the purpose of qualitative analysis only. Significance was indicated at *p* < 0.05; we have not used formal multiple comparisons correction to balance type I and type II errors as the core inferences of this paper were drawn from the multivariate analysis and not this supplementary univariate analysis.

## Results

### Ketamine robustly alters the whole-brain functional connectivity pattern

In order to determine the effect of ketamine on the pattern on degree centrality (DC), the ketamine condition was compared against both pre-infusion placebo (PLA + KET) and saline conditions which involved no active pharmacology. These comparisons yielded high classification accuracies reflecting significant differences in functional connectivity (Acc = 81.25 and 87.5 %, respectively, *p* < 0.003 and *p* < 0.001; Table 2).

**Table 2:** Results of PLA-SAL and PLA-KET classification using GPC

Contrast			Accuracy (%)	*p* value
Placebo (pre-Sal)	v	Placebo (pre-Ket)	68.75	0.52
Placebo (pre-Sal)	v	Saline	62.5	0.124
Placebo (pre-Ket)	v	Ketamine	81.25	0.003*
Saline	v	Ketamine	87.5	0.001*

Reported accuracies obtained with LOOCV and significance with (*N* = 1000) permutation testing

*Indicates results passing Bonferroni correction

Importantly, the classifier was unable to significantly distinguish between both the two pre-infusion conditions following administration of oral placebo (placebo pre-ketamine v placebo pre-saline) with a classification accuracy (68.75 %, *p* > 0.05) and conditions before and after a saline infusion (placebo (pre-saline) v saline, Acc = 62.5 %, *p* > 0.05) (Table 2). These findings indicate that within the context of our study design, we cannot discriminate between placebo (pre-saline) and saline conditions; this is akin to within-session stability. Furthermore, it was not possible to discriminate between placebo conditions which are akin to between-session stability.

**Fig. 3:**
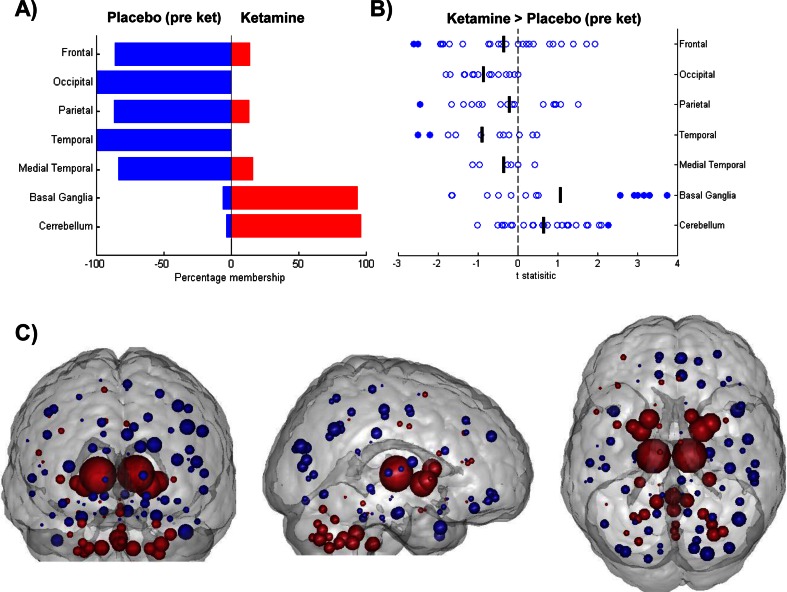
Results of univariate and multivariate analysis of node centrality for ketamine and placebo. **a** Distribution of classification weights within brain regions for the comparison of placebo (pre-ket; *Blue*) and ketamine (*Red*). *Bars* represent percentage membership for each class within the specified region. **b** Effect of ketamine on node DC values compared to placebo. One sample t-statistics for node changes (plabebo (pre-ket)—ketamine) summarised across brain regions. *Positive values* represent a ketamine-induced increase in DC in comparison to placebo. *Filled circles* signify significance, set at *p* < 0.05. **c** Visualisation of main effects of ketamine, g-map for classification between placebo (pre-ket; *Blue*) v ketamine (*Red*). *Nodes* correspond spatially to AAL regions and are shown with radii linearly scaled to indicate relative weighting. *Red nodes* exhibited increased centrality in t-maps, *blue nodes* exhibited decreasing centrality. 177 × 133mm (300 × 300 DPI)

Figure 3c illustrates the pattern of weights (g-map) driving the classification between the placebo (pre-ketamine) and ketamine conditions. Qualitatively, the pattern of node weights favouring the ketamine condition (depicted in red) is strongly represented in sub-cortical and cerebellar regions whereas the pattern of weights favouring placebo (pre-ketamine, depicted in blue) is predominantly cortical, especially in occipital, temporal, medial temporal and frontal cortical regions. A regional visualisation of the weight distributions is presented in Fig. 3a. This pattern of features was highly similar to that of the g-map of saline infusion compared to ketamine infusion, suggesting a similar effect driving the classification in both comparisons. Post hoc *t* tests performed directly on the DC values confirm the regions exhibiting increased DC, in relation to placebo, fit with the pattern of positive weights favouring ketamine, and vice versa for the pattern of negative weights. A summary figure illustrating the univariate change in DC values for each node within each region is shown in Fig. 3b.

### Pre-treatment with risperidone alters the ketamine-induced functional connectivity pattern

To investigate the effect of modulating the ketamine response by risperidone, classification was performed by comparing each of the RIS + KET sessions against both the placebo sessions (PLA + KET and PLA + SAL) as shown in Table 3.

**Table 3:** GPC classification using RIS + KET, LAM + KET, PLA + SAL and PLA + KET conditions, showing the effect of lamotrigine and risperidone pre-treatment on the ketamine-effected centrality

Contrast			Accuracy (%)	*p* value
Placebo (pre-saline)	v	Risperidone	75.00	0.007
Saline	v	Ris + ketamine	81.25	0.001*
Risperidone	v	Ris + ketamine	62.5	0.17
Ketamine	v	Ris + ketamine	81.25	0.001*
Placebo (pre-saline)	v	Lamotrigine	32.125	1
Saline	v	Lam + ketamine	93.75	0.001*
Lamotrigine	v	Lam + ketamine	87.5	0.001*
Ketamine	v	Lam + ketamine	43.75	0.872
Lamotrigine	v	Risperidone	62.5	0.154
Lam + ketamine	v	Ris + ketamine	93.75	0.001*

Reported accuracies obtained with LOOCV and significance with (*N* = 1000) permutation testing

*Indicates a results passing Bonferroni correction

**Fig. 4:**
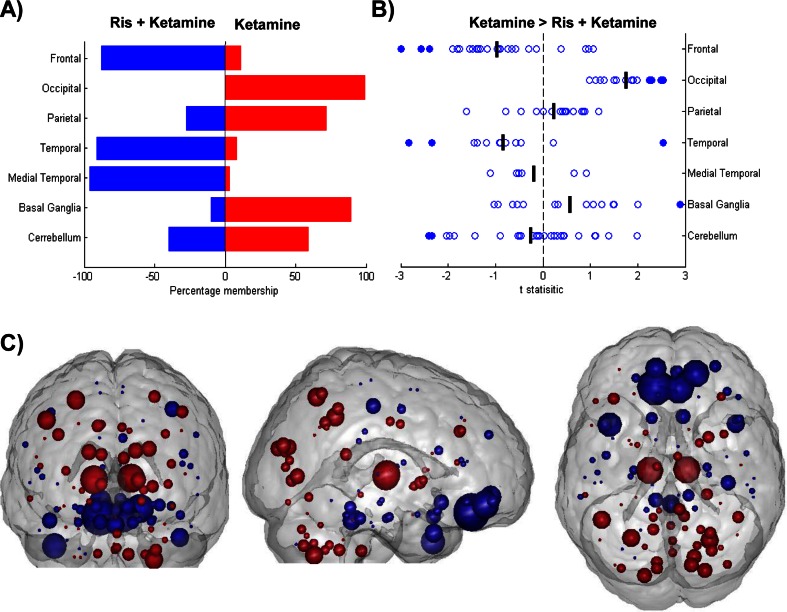
Results of univariate and multivariate analysis of centrality for ketamine pre-treated with placebo and risperidone. **a** Distribution of classification weights within brain regions for the comparison of Ris + ketamine (*Blue*) and ketamine (*Red*). *Bars* represent percentage membership for each class within the specified region. **b** Change in DC values due to risperidone pre-treatment of ketamine-induced changes. One sample t-statistics for node changes (defined as Ris + Ket—ketamine) summarised across brain regions. *Filled circles* signify significance, set at *p* < 0.05. **c** Effects of risperidone pre-treatment on ketamine-induced centrality changes, classification of Ris + ketamine (*Blue*) v ketamine (*Red*). *Nodes* correspond spatially to AAL regions and are representative linearly scaled to indicate relative weighting. *Red nodes* exhibit increasing centrality in t-maps, blue nodes, decreasing centrality. 173 × 134mm (300 × 300 DPI)

The DC pattern for the risperidone pre-treated ketamine condition was significantly discriminated from saline (saline v ris + ketamine, Acc = 81.25 %, *p* > 0.05) suggesting a dissimilar pattern of DC between conditions. Furthermore, the risperidone pre-treated ketamine condition was significantly discriminated from the placebo pre-treated ketamine condition (ketamine v ris + ketamine, Acc = 81.25 %, *p* < 0.001) indicating risperidone modulates the ketamine effect on DC; classification map is shown in Fig. 4c. This suggests a distinct risperidone state, different from both ketamine and saline. The spatial pattern of weights for ketamine pre-treated with risperidone was to some degree similar to ketamine pre-treated with placebo (Fig. 3c) with the notable exception of the weighting of nodes in occipital regions; this can best be observed in Fig. 4a. To investigate the univariate change in DC values, *t* tests were performed on the delta of ris + ketamine and ketamine conditions, Fig. 4b. Risperidone pre-treatment was observed to modulate ketamine-induced connectivity resulting in increased DC in the frontal and temporal cortices and decreased DC in the basal ganglia, occipital and parietal regions, when compared to placebo pre-treated ketamine.

Moreover, when comparing the pre- and post-ketamine infusion conditions for the risperidone pre-treated session, the classifier was unable to significantly separate these conditions (risperidone v ris + ketamine, Acc = 62.5 %, *p* > 0.05). The results of these three comparisons are all consistent with risperidone pre-treatment modulating the ketamine-induced functional connectivity pattern as measured by DC.

Whilst the results of these comparisons are consistent with risperidone modulating the ketamine-induced connectivity pattern, it is also possible that the classifications are driven by a common risperidone signature. That is, the main effect of risperidone may be present during both the risperidone and the ris + ketamine conditions. Thus, we propose that we are observing an interaction effect between risperidone and the ketamine response. This is in keeping with our hypothesis that risperidone has a substantial effect on the pattern of ketamine DC.

The risperidone pre-treated ketamine condition was classified using the GPC model trained using saline and ketamine classes (Table 4). Risperidone pre-treated ketamine was confused with both saline and ketamine conditions with chance level accuracies reported. A marginally higher accuracy was reported for the confusion with the ketamine condition than the saline.

**Table 4:**
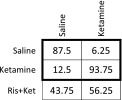
Confusion matrix for the classification of risperidone + ketamine using the GPC model obtained from training on saline and ketamine conditions

Testing was performed using leave-one-subject-out cross validation

### Pre-treatment with lamotrigine does not alter the ketamine-induced functional connectivity pattern

To investigate the effect of lamotrigine pre-treatment on ketamine-induced changes in functional connectivity, we compared both the lamotrigine conditions against the placebo post-infusion sessions (PLA + KET and PLA + SAL) as shown in Table 3.

It was not possible to discriminate lamotrigine from placebo (lamotrigine v placebo (pre-sal), Acc = 32.125 %, *p* > 0.05), suggesting similar patterns of DC in both conditions. There was no supportive evidence of a significant modulation effect of the ketamine-induced DC pattern by lamotrigine (ketamine v lam + ketamine, Acc = 43.75 %, *p* > 0.05). Furthermore, similar trends were observed when comparing the pre-infusion and post-infusion ketamine states for the placebo sessions (placebo (pre-ket) v ketamine, Acc = 81.5 %, *p* < 0.001) and lamotrigine pre-treatment (lamotrigine v Lam + ketamine, Acc = 87.5 %, *p* = 0.01). Taken together, our results suggest lamotrigine pre-treatment, at the administered dosage, does not have an effect on ketamine-induced DC patterns.

We were unable to distinguish between both lamotrigine and risperidone sessions when compared directly (lamotrigine v risperidone, Acc = 62.5 %, *p* > 0.05). However, a significant classification accuracy was observed for the comparison between post-ketamine conditions following a pre-treatment of lamotrigine and risperidone (lam + ketamine v ris + ketamine, Acc = 93.75 %, *p* < 0.001). This suggests both compounds differentially affect the pattern of ketamine-induced DC.

Ketamine plasma concentrations in the experimental sessions were correlated to participant posterior probabilities for the within session classification (pre-infusion vs. post-infusion). No significant correlation was observed at a level of *p* < 0.05. This suggests the observed patterns of DC do not linearly scale with the ketamine plasma concentrations.

### Ordinal regression

Ordinal regression was performed to investigate any potential attenuative effects of lamotrigine and risperidone on the ketamine-induced change in connectivity. Specifically, two analyses were performed with conditions rank ordered: (1) saline v (Ris + ketamine) v ketamine and (2) saline v (Lam + ketamine) v ketamine. As shown in Table 5, no ordinal trend was observed between saline and ketamine for (ris + ketamine) and (lam + ketamine) conditions. For configuration 1, saline is distinctly and accurately identified; however, the ketamine and the lam + ketamine classes exhibit confusion with each other. In configuration 2, saline and ketamine are well distinguished from each other; however, the ris + ketamine condition reports confusion with both conditions and is not well identified.

**Table 5:**
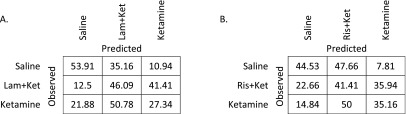
Confusion matrices for ORGP classification of (A) saline v lam + ketamine v ketamine and (B) saline v ris + ketamine v ketamine, no significant discrimination was observed for the pre-treated classes in relation to saline and ketamine, as indicated by the less than chance accuracy shown in the central cell (<33 %)

## Discussion

Using whole brain centrality metrics in a multivariate framework, we were able to show that ketamine induces a robust, predictable change in the functional connectivity pattern in the human brain, producing a shift from a cortically centred to a sub-cortically centred brain state. Risperidone pre-treatment significantly modulated the ketamine-induced centrality changes; however, lamotrigine did not. The distinct modulatory effects of these two compounds on ketamine-induced DC changes stands in contrast to their similar effects in attenuating the ketamine-induced BOLD amplitude changes.

### Ketamine infusion

Consistent with previous work, during a task-free acute ketamine challenge, a distributed pattern of functional connectivity changes were observed across the brain. On contrasting ketamine with placebo, nodes in the basal ganglia and cerebellum were predominantly weighted in favour of the ketamine group whilst nodes in the frontal, occipital, parietal, temporal and medial temporal lobe regions were weighted for the placebo state. Post hoc univariate tests confirm the directionality of these effects, revealing decreasing cortical centrality and an increase in centrality of the cerebellum and basal ganglia in the ketamine state relative to the control.

Previous studies have revealed findings consistent with observations from this study. An overall increase in “global connectivity” as a result of ketamine infusion was observed in healthy volunteers (Driesen et al. 2013). Regions exhibiting the greatest increase in this global connectivity for ketamine included the thalamus, parietal regions and cerebellum. Whilst our analysis is multivariate, it is noteworthy that the same regions are present in the pattern of features describing ketamine and have increased degree centrality in relation to placebo. Our analysis extends these previous works, revealing the pattern on changes across the whole brain between groups.

Ketamine has also been reported to strongly affect the connectivity of cerebellar, visual, auditory, somatosensory and subcortical regions in relation to pre-defined networks of interest (Niesters et al. 2012). The cerebellum was shown to have increased connectivity in relation to visual networks as well as the intra-network connectivity of the visual cortex when under the influence of ketamine. Furthermore, the thalamus, PFC, temporal cortex and cingulum were shown to have decreasing connectivity in relation to the auditory and somatosensory network.

The multivariate results presented here provide an alternative perspective on the effect of acute ketamine on whole-brain connectivity. The task-free connectivity organisation of the brain is radically altered by acute ketamine with both increases and decreases in the centrality pattern of the brain. This is supported by previous studies demonstrating ketamine administration can cause long-range decoupling of neural population activity in mouse neocortical slices and has been shown to cause both increases and decreases in connectivity, e.g. (Dawson et al. 2013). We can summarise the observed effects of acute ketamine in humans as a shifting of the pattern of connectivity from a cortically centred state to a sub-cortically centred state. It is possible that cortical regions have a global reduction in connectivity with the rest of the brain, resulting in a reduction in the connectivity of cortical hubs; alternatively, it is possible that dominant regional couplings are reduced, resulting in a more equally distributed connectivity. Both mechanisms would result in the DC pattern observed under ketamine. The presented methodology is not designed to identify changes in individual connections; instead, we reveal consistent patterns of DC change across the whole brain which reveals a subcortical contribution for the ketamine connectivity pattern.

The observed pattern of disconnectivity of the cortical hubs is consistent with many of the experiential effects observed with ketamine; interruption of connectivity between cortical hubs would be predicted to result in a reduction contextual processing, impaired memory, spatial representation and sensorimotor processing. Whilst not a phenocopy of the disorder, ketamine has been used as pharmacological model for schizophrenia which has been shown to exhibit similar alterations in connectivity, notably a reduction in high DC cortical hubs (Rubinov et al. 2009) and wide spread disconnections across the brain (Liang et al. 2006; Lynall et al. 2010).

The main effects of ketamine at sub-anaesthetic doses are typically attributed to its blockade of the NMDA receptor (Driesen et al. 2013). It has high affinity for NMDA and D_2_ receptor sites as well as a slightly lower affinity for the 5-HT_2A_ receptor, muscarinic and opioid receptors although these are thought to have notable effects at higher doses only (Hirota et al. 1999; Narita et al. 2001). A possible mechanism by which ketamine effects connectivity changes is through interruption of NMDAR-mediated transmission, namely the known cortical microcircuit effects where parvalbumin-positive GABAergic interneurons disinhibited by ketamine (Homayoun and Moghaddam 2007). Microdialysis confirms the NMDA antagonists PCP and MK-801 increase extracellular GABA levels. These interneurons inhibit pyramidal cells and synchronise their oscillations (Homayoun and Moghaddam 2007). The disinhibition of these GABAergic interneurons may result in impaired pyramidal synchrony and hence in decreased connectivity of cortical nodes, leading to the observed pattern of centrality. It is suggested that the lateral posterior pulvinar in the thalamus may strongly contribute to pyramidal synchrony (Shumikhina and Molotchnikoff 1999) which may explain its inclusion in the pattern for ketamine exhibiting increased centrality. Alternatively, if ketamine increases cortical connectivity globally whilst reducing strong regional connections, this may also result in increased centrality of the basal ganglia and cerebellum whilst reducing cortical centrality.

The radical alteration of the pattern of centrality during a task-free ketamine infusion has not been demonstrated previously and suggests a complex network reorganisation underpinning the effect of ketamine, even at the low dose administered in this study. This reorganisation is a plausible candidate for the variety of effects produced by ketamine, such as perceptual distortion (via parietal and visual regions as well as cerebellar and basal ganglia timing nodes), cognitive disorganisation (via fronto-patietal nodes) and anhedonia (via striatal and ventromedial nodes). The important contribution of the current study is to inform on the reorganisation of brain networks by ketamine and to what degree these can be pharmacologically modulated by different pharmacological mechanisms. In order to facilitate multiple administrations of ketamine, doses were kept relatively low; as such, we did not observe any strong and consistent subjective effects. Furthermore, the sparseness and inconsistencies of participant subjective responses, which were collected, prohibited investigation of correlation between functional connectivity effects and subjective ratings. Replication using higher doses producing stronger subjective changes in all participants would be important and also allow testing of the relationship between the multivariate changes in network patterns with the profiles of changes in subjective reporting of drug effects.

### Lamotrigine treatment

Lamotrigine causes a reduction in glutamate release primarily through Na^+^ channel modulation; we hypothesised that this would partially counter the downstream glutamatergic effects of ketamine, thus attenuating any ketamine-induced changes in functional connectivity. Indeed, it has been demonstrated that lamotrigine pre-treatment attenuates the ketamine BOLD effect (Deakin et al. 2008; Doyle et al. 2013a; b). Furthermore, it has been suggested that ketamine’s effect on functional connectivity may reflect glutamatergic modulation (Duncan et al. 2011). In contrast to our previous findings on the ketamine-induced BOLD signal, we found no significant effects of lamotrigine on the pattern of DC in the brain. The administered dose of lamotrigine (300 mg) is clinically effective and the data analysed is the same as that used in our previous study (Doyle et al. 2013a; b) where clear effects on the local BOLD signal were demonstrated. Instead, our combined results show that lamotrigine induces an amplitude change in signal rather than affecting the coupling between regions; although we cannot preclude more subtle, localised changes in connectivity which our method which interrogates whole brain DC patterns would be insensitive. This lack of DC modulation by lamotrigine is further supported by the ordinal regression of saline, lamotrigine + ketamine and ketamine conditions, where we did not observe an ordinal progression between classes. This is consistent with our conclusion that lamotrigine does not attenuate the ketamine-induced pattern of DC. The strong confusion between the lamotrigine + ketamine and ketamine conditions as observed using binary classification whereby the discrimination between the lamotrigine + ketamine conditions was not significant (Table 3) and for ordinal regression (Table 5) whereby ~41 % of the lamotrigine + ketamine cases were classified as ketamine.

These results have important mechanistic implications for the ketamine-induced connectivity response and support the conclusion that lamotrigine pre-treatment has a similar effect to placebo on ketamine-induced DC. Furthermore, since lamotrigine, which block glutamate release, does not significantly modulate the ketamine-induced effects then this suggests that the observed pattern of hub connectivity induced by ketamine reflects NMDAR antagonism per se rather than downstream glutamatergic effects at non-NMDA sites. Instead, this would favour the NMDA blockade of ketamine as responsible for the observed connectivity changes. This proposal could be confirmed in preclinical experiments using specific NMDA subunit antagonists and non-NMDA antagonists for the AMPA and kainate receptors.

### Risperidone treatment

Risperidone (2 mg) is expected to achieve high occupancy of 5-HT_2A_ and D_2_ receptors (Nyberg et al. 1999) and reduces glutamate levels downstream through 5-HT_2A_ antagonism. Furthermore, risperidone has also been reported to potentiate NMDAR function directly (Konradsson et al. 2006). Given this profile, we expected that risperidone pre-treatment would attenuate the pattern of DC induced by ketamine.

Indeed, our observations indicate that, in contrast to lamotrigine, risperidone modulates the effect of ketamine resulting in a pattern of DC distinct to both ketamine and saline. The classification maps for ketamine compared to saline and ketamine compared to ris + ketamine also reveal a strong overlap in the spatial distribution, but not amplitude, of weights with the exception of occipital regions. This is consistent with risperidone pre-treatment reversing the ketamine-induced node strength changes. Comparing the pre- and post-infusion conditions for ketamine pre-treated with risperidone reveal risperidone effects some more subtle changes that did not achieve statistical significance. Direct comparison of the ketamine and ris + ketamine states reveal strong effects of the risperidone pre-treatment on the ketamine-induced pattern of DC which appear to oppose the ketamine effect. Furthermore, the ORGP results suggest risperidone does not attenuate the ketamine DC response in an ordinal manner; instead, the combined results support the conclusion that risperidone may have an opposing effect on the ketamine response.

We theorise that it is the twofold mechanism of risperidone acting upon ketamine that is responsible for the observed DC effects. It is likely that the potentiation of the NMDAR is the primary mechanism due to the dissimilarity in resultant effects between risperidone and lamotrigine pre-treatment. Our results are suggestive that risperidone may interact with, and in opposition to, the ketamine resulting in a pattern of DC dissimilar to that of ketamine; the dissimilarity from the saline state and ordinal regression results preclude a linear attenuation effect of risperidone on ketamine. These observations may be predominantly due to serotonergic effects; however, further work is required using a compound with selective 5-HT_2A_ binding.

In addition to the NMDA receptor, ketamine also has affinity for other receptors including dopamine, D2 and opioid receptors, particularly at high doses, effects can also be elicited through downstream serotonergic and muscarinic receptors (Hirota et al. 1999; Narita et al. 2001). Whilst we cannot preclude effects at other receptors contributing to our findings, we would favour a major contribution from glutamatergic effects. The opposing effects of ketamine and risperidone in the striatum may conceivably be related to their opposing effects at the D2 receptor, where risperidone acts as an antagonist and ketamine an agonist. The use of selective compounds would be required to understand the contribution of individual receptor types to the observed pattern of effects.

### Stability of pattern recognition method in the absence of pharmacological intervention

Importantly, our methodology did not detect changes in connectivity pattern when no pharmacological stimulus was applied. Comparisons between pre-infusion scans from the two oral placebo sessions (PLA + SAL vs. PLA + KET), as well as between the pre- and post-infusion scans from the saline infusion session (PLA + SAL), both revealed no significant differences with classification accuracies approximating chance level. This test-retest stability provides confidence in our interpretation of the effects of ketamine and its modulation by risperidone and lamotrigine as reflecting the pharmacological action of these interventions.

### Limitations

The results presented here provide an informative insight into the mechanisms of effect of ketamine on the human brain; however, the limitations of this analysis must also be considered. The use of graph theory combined with machine learning is a novel approach; it provides a principled means of investigation pharmacological compounds on the human brain. The use of centrality, whilst providing an intuitive measure of regional connectedness and providing an elegant solution to the interpreting spatial patterns of discrimination, prohibits investigation into specific regional coupling. As such, this methodology is insensitive to subtle changes in regional coupling which may be induced by the administered compounds. Univariate methods (Niesters et al. 2012) are available for testing hypotheses about specific connections, but these might also be more sensitive to non-pharmacological effects. Our choice of examining the patterns of change across the brain was motivated by the widespread expression of NMDA receptors.

The use of a single connectivity metric and temporal scale is a further limitation. The use of correlation allows for the identification of positive and negative synchronous relationships between regions, however, is naive to phase-delayed and non-linear relationships between regions which are likely to exist in a complex biological system such as the brain. By comparing the results of analysis using both correlation and a phase insensitive measures such as coherence, it may be possible to infer the effects of ketamine on BOLD signal synchronisation between regions. It is likely that analysis performed using different windowing scales would provide further insight into the effects of ketamine on the dynamics of connectivity in the human brain.

The use of degree centrality provides an initial investigation into the connectivity effects of ketamine in the brain. However, in order to generate a complete view of the connectivity effects of ketamine, a range comprehensive range of measures should be compared. Several graph theory metrics exist informing differential aspects of function connectivity; however, many of these measures are highly correlated and present challenges in biological interpretation. Further investigation is required in order to identify an optimal set of orthonormal connectivity measure suitable for use in connectivity analysis which provides a broad overview of connectivity effects.

## Conclusions

We have demonstrated a robust pattern of acute connectivity changes resulting from administration of a sub-anaesthetic dose of ketamine, comprising a reduction in connectivity of cortical nodes and an increase in DC of nodes in the basal ganglia and cerebellum. Importantly, our methodology detected no changes in the connectivity pattern for paired comparisons involving no pharmacological stimulus.

Furthermore, pre-treatment with risperidone, but not lamotrigine, resulted in a strong modulation of the ketamine-induced hub changes. This likely reflects the distinct mechanisms of the two compounds and suggests that the observed changes in connectivity are more likely a result of NMDA blockade and possible serotonergic modulation rather than purely modulation of glutamate release. The differential modulatory effects of these two compounds are in contrast to the finding that both compounds similarly attenuate the ketamine-evoked BOLD signal amplitude changes (De Simoni et al. 2013; Doyle et al. 2013a). This connectivity analysis provides distinct information regarding the effects of neurologically active compounds and inform on the neural circuit correlates of specific pharmacological mechanisms.
